# Animal Behaviour Packs a Punch: From Parasitism to Production, Pollution and Prevention in Grazing Livestock

**DOI:** 10.3390/ani14131876

**Published:** 2024-06-25

**Authors:** Lesley A. Smith, Naomi J. Fox, Glenn Marion, Naomi J. Booth, Alex M. M. Morris, Spiridoula Athanasiadou, Michael R. Hutchings

**Affiliations:** 1 Animal and Veterinary Sciences, Scotland’s Rural College (SRUC), West Mains Road, Edinburgh EH9 3JG, UK; naomi.booth@sruc.ac.uk (N.J.B.); morrisa32@cardiff.ac.uk (A.M.M.M.); spiridoula.athanasiadou@sruc.ac.uk (S.A.); mike.hutchings@sruc.ac.uk (M.R.H.); 2 Biomathematics and Statistics Scotland (BioSS), Kings Buildings, Edinburgh EH9 3FD, UK; glenn.marion@bioss.ac.uk

**Keywords:** behaviour, parasite, grazing, methane, trade-off, ruminant, growth, efficiency, resistance

## Abstract

**Simple Summary:**

Individual animal behaviours can often drive broader scale effects with potentially unexpected knock-on consequences. However, animal behaviour is often neglected in standard approaches to infection control, food security and climate change adaption/mitigation. To highlight the importance of animal behaviour, we describe the animal behavioural determinants of gastrointestinal parasitism and its knock-on consequences for grazing livestock production and pollution. We then consider how we might exploit animal behaviour to mitigate the consequences of parasitism. Grazing livestock face a nutrition versus parasitism trade-off and use behavioural rules of thumb when making grazing choices that determine their intake of nutrients and parasites, i.e., production efficiency. Parasitism is often associated with reduced food intake, a change in grazing decisions and slower growth. As methane is a by-product of rumination and a greenhouse gas (GHG) contributing to climate change, parasitism is associated with greater methane production from ruminant livestock. Parasitism results in animals on-farm for longer and more methane per kg food intake, creating a double hit of more methane produced for longer. However, the parasitism-induced sickness behaviours in grazing livestock can be detected and thus potentially exploited for control purposes. Activity sensors can detect parasite-induced behavioural change within a few days of infection, and thus offer a potential way to identify animals for treatment before production and pollution costs are realised. We conclude that livestock host x parasite interactions are at the centre of the global challenges of food security and climate change, and that understanding livestock behaviour can contribute to solving both.

**Abstract:**

Behaviour is often the fundamental driver of disease transmission, where behaviours of individuals can be seen to scale up to epidemiological patterns seen at the population level. Here we focus on animal behaviour, and its role in parasite transmission to track its knock-on consequences for parasitism, production and pollution. Livestock face a nutrition versus parasitism trade-off in grazing environments where faeces creates both a nutritional benefit, fertilizing the surrounding sward, but also a parasite risk from infective nematode larvae contaminating the sward. The grazing decisions of ruminants depend on the perceived costs and benefits of the trade-off, which depend on the variations in both environmental (e.g., amounts of faeces) and animal factors (e.g., physiological state). Such grazing decisions determine the intake of both nutrients and parasites, affecting livestock growth rates and production efficiency. This impacts on the greenhouse gas costs of ruminant livestock production via two main mechanisms: (1) slower growth results in longer durations on-farm and (2) parasitised animals produce more methane per unit food intake. However, the sensitivity of behaviour to host parasite state offers opportunities for early detection of parasitism and control. Remote monitoring technology such as accelerometers can detect parasite-induced sickness behaviours soon after exposure, before impacts on growth, and thus may be used for targeting individuals for early treatment. We conclude that livestock host x parasite interactions are at the centre of the global challenges of food security and climate change, and that understanding livestock behaviour can contribute to solving both.

## 1. Introduction

The fundamental role of behaviour in disease transmission was highlighted by the SARS-CoV-2 pandemic, where changes in behaviour operating at the individual level (e.g., due to cycles of lockdown) were seen to change the epidemiological patterns of infection seen at the population level [1]. Similarly, consequences of behaviour can be found in animal disease ecology, where animal behaviour is the fundamental biological mechanism for disease-transmission events. In this review we consider the importance of animal behaviour in the disease ecology of gastrointestinal parasites in grazing livestock. In the case of gastrointestinal parasitism, transmission is often via the faecal oral route, and the behaviour of grazing livestock in relation to environmental distributions of faeces drives transmission. To highlight the importance of animal behaviour, we describe the animal behavioural determinants of gastrointestinal parasite transmission and the resultant patterns of parasitism in grazing livestock that often lead to further consequences in the farming systems affected.

Gastrointestinal parasitism is a ubiquitous and pervasive challenge to grazing livestock. Whilst often found at sub-clinical levels, parasitism affects animal growth and thus production efficiency [2]. The world population is growing and demand for livestock products is forecast to double by 2050 [3]. Meeting the nutritional needs of a growing population will require sustainable intensification of livestock production and a need to reduce production losses such as those created by gastrointestinal parasitism. Furthermore, ruminant livestock production efficiency impacts enteric methane production and thus greenhouse gas (GHG) pollution [4]. Climate change caused by GHG emissions is expected to further impact agricultural production, exacerbating the threat of global food insecurity [5]. This puts ruminant livestock health at the centre of a number of global challenges, namely, food security and climate change. Here we take a bottom-up approach, characterising and quantifying how animal behaviour can impact parasite transmission and patterns of infection, and then track its knock-on consequences on livestock production and pollution. Despite its crucial role in disease systems, animal behaviour is often overlooked when designing sustainable agricultural practices or planning robust adaptations to the anticipated effects of climate change. We propose that incorporating animal behaviour into disease mitigation strategies could enhance food security and address climate change challenges.

## 2. Parasitism and the Impact on Production

### 2.1. Gastrointestinal Parasitism—A Consequence of Animal Behaviour

Lifecycles of many gastrointestinal parasites (e.g., nematodes) consist of developmental stages within the host that produce eggs that are excreted in faeces into the environment, where they develop into infective stage larvae that when ingested complete the lifecycle. Grazing livestock cannot detect the parasites on pasture [6], and thus use faeces as a cue to the presence of parasites [7]. As such, grazing livestock will avoid grazing faecal contaminated pasture, preferring to graze non-contaminated pasture [8,9]. Importantly, fresh faeces are avoided most strongly and the strength of avoidance declines as the faeces decomposes [7,10]. In cases where the infectious agent does not survive for extended periods outside of the host in the environment, such as bacterial and viral pathogens, the strongest avoidance of fresh faeces results in livestock avoiding the greatest concentrations of infectious agents, e.g., grazing cattle avoiding *Mycobacterium bovis* in badger faeces [11]. In contrast, gastrointestinal parasites deposited as eggs in the faeces can take many weeks to develop into infective stage larvae [12], and so the strength of the faecal avoidance cue has diminished by the time the faeces represent the greatest infectious threat [13]. Faecal avoidance and the timing of parasite development in the environment creates a nutrition versus parasitism trade-off, the costs and benefits of which change over time [14]. The benefits of the trade-off are two-fold: (1) the initial strong avoidance of faeces-contaminated swards relative to non-contaminated swards creates patches of tall sward, that offer the opportunity for greater nutrient intake rate, as sward height is positively correlated with ingestion rate [15,16], and (2) nutrients (e.g., nitrogen) leach from the faeces in to the surrounding sward, fertilising it, creating patches of relatively nutrient rich sward [17]. The costs of the trade-off relate to the number of infective stage larvae on the sward. The infective stage larvae migrate from the faeces via water films on to the sward, with the highest concentrations often found at the top and bottom of the sward [18]. The costs and benefits of herbivore grazing decisions in relation to the nutrition x parasitism trade-off have been quantified by [19]. Soay sheep were introduced into a field with homogeneous sward height and over time the sward structure became increasingly heterogeneous through avoidance of grazing areas contaminated with faecal deposits, leading to a gap (short, grazed areas) and tussock (tall faecal contaminated areas) structure. By week 12 of the experiment, sward pluck samples (simulated bites) suggested that the tussock patches offered the grazing sheep 1.5 times greater dry matter and nitrogen intake. However, tussocks also contained 5.5 times greater infective stage parasite larvae compared to the relatively short gap patches. Thus, the initial faecal avoidance behaviour created the nutrition vs parasitism trade-off for subsequent grazing decisions.

Parasite transmission is a consequence of the grazing decisions of an animal in relation to this patchwork distribution of nutrients and parasites in grazing environments [10]. Grazing livestock have behavioural skills to navigate this heterogeneous environment that can be seen as behavioural rules of thumb; avoid faeces, select nitrogen rich swards (darker) and select taller swards [20]. The nutrition versus parasitism trade-off in grazing systems is highly dynamic, as the sward height, sward nutrient content and the number of infective stage parasites change over time [10,14,21]. Furthermore, the background environmental conditions affect the relative costs and benefits e.g., the fertilising effect of the faeces on the size of the benefit offered to grazing livestock will be greater in nutrient poor systems [22]. Similarly, the management of livestock affects the development of the trade-off e.g., stocking rates [23]. The visual cue (sward height and darkness), used to determine the potential benefits of the trade-off, can be detected from a greater distance than the olfactory cue (presence of faeces) used to determine the risk of infection. This leads to a two-stage contact process, where initially grazing livestock approach the faecal contaminated, relatively tall and nutrient rich patches of sward, only to potentially reject them for grazing once the faeces is detected [24]. Capturing this behavioural interaction with the nutrition vs parasitism trade-off within simulation modelling frameworks has shown its potential impact on animal, parasite exposure [20], forage intake rate [25], and parasite burdens [26], including under future climate change scenarios [27].

### 2.2. Adaptive Animal Behaviour Driving Parasite Transmission Dynamics

**Figure 1:**
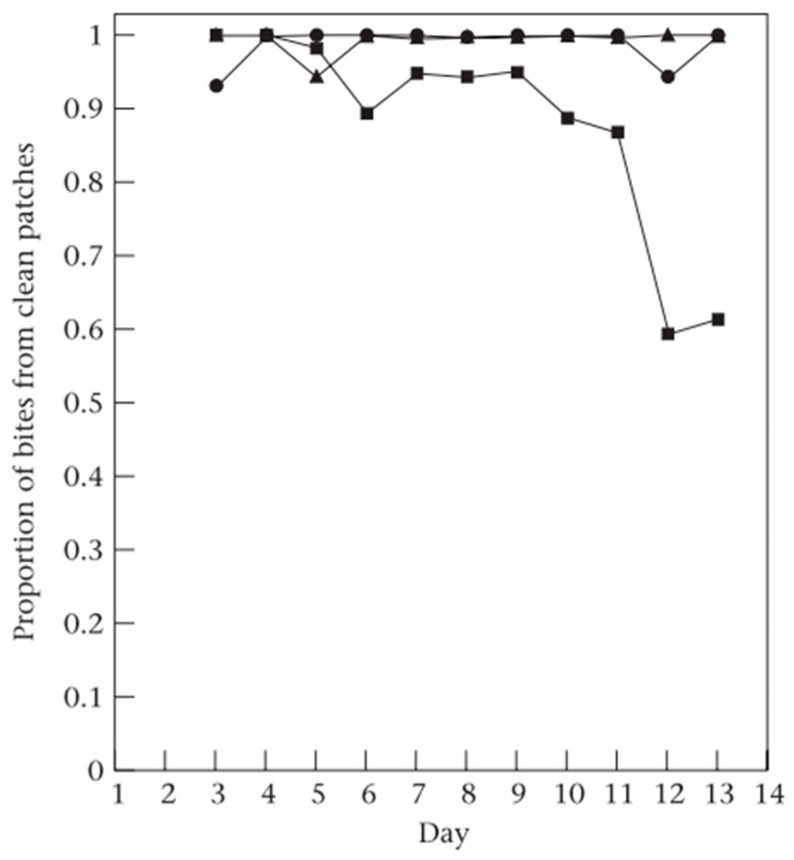
Effect of animal parasite status on the diet selection of sheep grazing a gap (clean uncontaminated patches) and tussock (faecal contaminated patched) mosaic. Proportion of bites taken from clean patches by sheep immune to *Teladorsagia circumcincta* (■), parasitised with *T. circumcincta* (▲) and non-parasitised (●) (graph created by Michael R. Hutchings, from [14]).

The relative costs and benefits of the nutrition versus parasitism trade-off, and in turn the grazing decisions which determine nutrient and parasite intake are affected by the animal’s physiological state (e.g., feeding motivation, immune state) [28,29]. When faced with the trade-off, parasitised animals can increase their faecal avoidance, relative to their non-parasitised counterparts (uninfected animals taking 1.5 times more bites from faecal contaminated swards) [7], preferring to avoid additional parasite exposure over the nutritional benefits offered. The intensity of the parasite burden has also been shown to affect herbivores grazing decisions in relation to the trade-off, with low parasite burden sheep taking 1.3 times more bites from faecal contaminated tussocks relative to high parasite burden animals [10], enabling them to take advantage of the nutritional benefits associated with the trade-off. This suggests that herbivore hosts are not only sensitive to the presence of gastrointestinal parasites they harbour, but also the degree of parasitism and alter their behaviour in response to this. Similarly, parasite immune animals, which are at less risk from parasites, reduce their proportion of bites from clean uncontaminated patches and increase their proportion of bites from faecal contaminated patches (Figure 1), to take advantage of the nutritional benefits of the trade-off [14]. The nutrient intake requirements of animals also affect the relative strength of the costs and benefits of the trade-off, with restricted fed, highly feeding motivated animals taking a 2.3 times greater proportion of bites from tall faecal contaminated swards relative to ad libitum fed sheep, accepting a greater risk of parasitism to take advantage of the increased nutrition of the trade-off [29].

**Figure 2:**
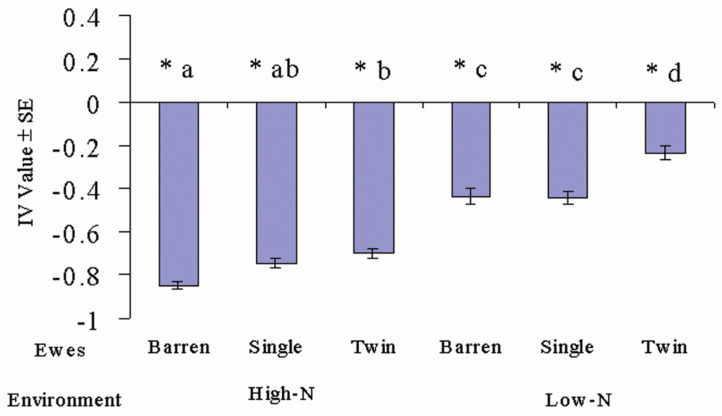
Effects of nitrogen and the ewe’s physiological state (barren ewes; ewes suckling a single lamb and ewes suckling a twin lamb) on the selection of faecal-contaminated tussocks while grazing. Figures are mean Ivlev’s electivity index (IV) values ± SE. This is an index of diet selection that compares the proportion of the food type selected relative to the proportion of that food type available in the environment. IV values show tussock selection in relation to tussock availability (i.e., translating bites from tussocks into selection (+1 IV values) or avoidance (−1 IV values)). Asterisks (*) denote significance from zero. Letters denote significant differences between treatments. IV values demonstrate that ewes maintain a degree of avoidance to faecal-contaminated tussocks, but the nutritional environment and reproductive effort of the ewes affect the degree of avoidance (graph created by Lesley A. Smith, from [22]).

However, in livestock production, animals experience management practices, which can impact the cost and benefits of the trade-off. For example, in nutrient-poor environments such as extensive grasslands, nutrient leaching from faeces may significantly increase the nutritional value of the faecal-contaminated tussocks [17], which may impact a grazing herbivore’s decisions in these environments. Other management practices such as lactation, increase the energetic demand and nutrient requirements of animals, whilst also making them more susceptible to parasites due to a periparturient relaxation in immunity [30]. The impacts of the nutritional environment and lactation on sheep grazing decisions in relation to the trade-off were investigated by [22] and it was demonstrated that peri-parturient and lactating animals reduce their faecal avoidance and increased their risk of parasitism. Furthermore, lactating sheep grazing in low-nutrient environments further reduced their avoidance of tussocks (Figure 2). Thus, lactating sheep accepted the increased risk of parasitism to prioritise nutrient intake, and invest in their offspring [22]. However, this grazing choice often results in immuno-compromised peri-parturient females that are heavily infected with parasites further increasing the environmental burden of parasites and exposing their parasite-naïve offspring [31]. It is during the offspring’s transition from susceptibility to immunity that much of the impact of parasites on livestock production efficiency is seen.

### 2.3. Production Impacts of Parasitism

To tackle global challenges that hinder food production sustainability, such as antimicrobial resistance, climate change and maintenance of biodiversity, it is critical that the rate of livestock productivity and profitability can be maintained, if not improved. Gastrointestinal parasitism caused by nematodes and trematodes has high economic importance worldwide. In a recently published assessment, the annual cost of helminth infections in Europe was estimated at approximately EUR 1.8 billion [32]. Approximately 80% of this cost was attributed to production losses, including low carcass weight and milk production, which highlights the true impact of helminth infections across production systems. To achieve sustainable food production for an ever-growing human population, which is estimated to reach 9.7 billion people by 2050 [33], there is a requirement to adopt more efficient and sustainable production strategies whilst achieving climate change mitigation. Whilst parasitism can be seen to have several impacts on the host [34], a key behavioural component directly impacting on livestock production efficiency is anorexia, a voluntary reduction in food intake, with consequences for performance (e.g., growth) and feed conversion efficiency.

### 2.4. Parasite-Induced Anorexia

The voluntary reduction in food intake in animals, anorexia, is a characteristic of many bacterial, viral and parasitic infectious diseases (see reviews [35,36,37]). For gastrointestinal parasites the impairment is due to the increased endogenous loss of protein, but also from increased damage of gut tissue and mucoprotein secretion [38,39,40]. Gastrointestinal parasitism can also cause 20–50% reductions in daily dry matter intake (g DMI/day) [34,41] and up to 50% reduction in growth performance for the same level of feed intake, in subclinical infections [42]. Genetic susceptibility to pathogens can affect the level of anorexia observed; periparturient ewes genetically resistant to helminth nematodes reduced their feed intake by 20% when infected with *T. circumcincta*, whereas susceptible ewes reduced their intake by 32% under the same infection and nutritional conditions [43]. A reduced feed intake during an energy-demanding parasite infection can seem counterintuitive [34]. Parasite-induced anorexia is considered a first sign of preclinical disease in a large array of hosts, from insects to mammals; many theories have been developed to justify its occurrence. One hypothesis is that it is an adaptive, although costly, response to parasitism by the host to reduce further exposure to parasites [44]. It also has an evolutionary link with host’s resistance to infections (i.e., via enhancing immune responses) as it potentially reduces costs associated with resource acquisition, digestion and possibly detoxification, which are functions that can interfere with and penalise immune responses to pathogens. In sheep infected with *Trichostrongylus colubriformis*, anorexia does not occur until 3–4 weeks after ingestion of larvae, which coincides with the establishment of mature worms in the small intestine [45]. The timing of this occurrence indicates that anorexia may not be a behavioural mechanism that affects the host during the acquisition of immunity to worms, i.e., the early stages of immune responses, but rather it presents itself at the later time of the expression of immunity. Gastrointestinal parasite-induced anorexia often resolves after the parasites have been removed from the host either through a successful mounting of the host immune response [45] or administration of antiparasitic medication. Importantly, the recovery in appetite occurs before the gastrointestinal tract has had time to recover from the caused pathology (see review [34]), indicating that anorexia is not attributed to the damage caused in the gastrointestinal tract by the pathogen. It is important to note that even in the presence of an effective antiparasitic treatment, partly due to the damage caused by the parasites and direct consequences on digestibility, animals require additional time on-farm to achieve the required growth. It has been elegantly demonstrated that *T. colubriformis* infection resulted in a 20% drop in feed intake in sheep and a significant reduction in weight gain by 50%, which was alleviated following anthelmintic administration by about 25% [46]. This increased presence of animals on pasture to achieve the compensatory growth required to achieve finishing bodyweight will consequently have long-term impacts on pollution [47].

## 3. The Impacts of Parasitism on Pollution

### 3.1. Ruminants and GHG Production

In ruminants, a proportion of food energy is lost to the atmosphere as methane, a key GHG. During the fermentation process, food matter is broken down by microorganisms in the rumen into products that can be easily absorbed by the rumen and small intestine. Methanogens then convert the resultant hydrogen and carbon dioxide into methane, which is released as a gas into the atmosphere through eructation. Methane production in ruminants is closely related to dry matter intake (DMI) [48].

Methane production represents a production loss as this proportion of the gross energy intake in the feed is lost to the environment rather than being used for growth and/or milk production. Depending on the feed, livestock methane emissions could represent a 2–15% loss of gross energy from feed [49]. Agriculture is a major contributor to GHG emissions, with enteric methane emissions from ruminant livestock contributing an estimated 16% of global methane emissions [50]. Furthermore, for individual countries, agriculture and specifically livestock methane emissions can contribute significantly to their gross emissions. For example, in New Zealand the agricultural sector is the country’s largest source of emissions (53.2%), with 22.4% and 11.4% of gross emissions being attributed to methane from dairy cattle and sheep, respectively [51]. Methane gas has 28 times more global warming potential (GWP) over 100 years compared to the same amount of CO_2_ [52]; however, methane has a relatively short life in the atmosphere (12 years) compared to carbon dioxide (300 to 1000 years) [53]. Average global temperatures have reached or exceeded 1.5 °C above pre-industrial temperatures and breaching the long-term IPCC global temperature targets seems inevitable [54]. Due to their relatively high GWP and low atmospheric lifespan, methane reductions offer a near-term solution to meeting emission reduction targets. Thus, to achieve national emission reduction targets, incorporating a reduction in livestock methane emissions could be effective. Ruminant methane intensity is driven by feed intake, the activity of the microbiome and feed conversion efficiency. As disease impacts on these core processes, animal health will affect GHG emissions from livestock.

### 3.2. Livestock Health and GHG Emissions

Several studies have considered the effects of parasitism on livestock GHGs. Earlier studies explored the indirect effects, looking at the interactions between production efficiency and methane pollution, e.g., reduced productivity leading to larger flocks or herds to meet production targets and increased cull and replacement rates all contributing to more animals being alive and on-farm for longer if disease is present [55,56]. Later work investigated the direct effects, measuring the impacts of parasitism on GHG emissions using respiration chambers [47,57].

Upon evaluating 12 endemic livestock diseases, [56] found that disease control of each of the evaluated pathogens would result in a reduction in GHG intensity (GHG emitted per unit of meat/milk). This study concluded that the disease with the greatest GHG abatement potential in sheep was parasitic gastroenteritis—an infection caused by parasitic nematodes. Their analysis, based on indirect effects on emissions such as production efficiency, mortality, fertility and replacement rates, concluded that parasite control could lead to a 9% reduction in emissions intensity across all sheep production systems [56]. This echoes the findings of [55], who found that different parasite treatment regimes in lambs affected their time to reach slaughter weight, and that waiting for evidence of clinical signs before treating led to a significant decrease in production efficiency, increased time on pasture to reach slaughter weight and therefore a predicted increase in GHG emissions. In these studies, the impacts of animal health on GHG emissions are mainly calculated based on the additional time animals are on-farm (i.e., alive and eructating) due to slower productivity when infected [55,56] and changes in mortality and fertility [56]. These studies had the potential to underestimate the mitigation potential of disease control as they did not consider potential direct effects of parasite infection on methane production. Respiration chambers have since been used to measure methane emissions in sheep during a *T. circumcincta* infection [47,57]. The first study in periparturient ewes resulted in increased GHG emissions during parasitism that were largely attributed to feed efficiency [57]. A further study in growing lambs measured the impacts of parasitism on methane yield (gCH_4_/kgDMI) at the point where the adult parasite worm burden was near its peak [47]. A parasite-induced anorexia in the infected animals was observed, and this reduced food intake led to a reduction in absolute amount of methane from infected animals on a daily basis. When feed intake was accounted for, the parasitised animals showed a 33% increase in methane emissions per kilogram of dry matter intake (DMI) compared to the non-parasitised control groups [47]. The direct effect seen in this study is in addition to all the indirect effects previously reported [55,56]. As discussed earlier, parasitised lambs have decreased production efficiency, gaining less weight for every kg of feed intake. For every bite of feed ingested, parasitised animals are gaining less weight and emitting more methane than uninfected ones [47], these parasitised animals are also maintained on-farm for longer to enable them to overcome the reduced growth rates and reach commercial slaughter weights [55]. These elements combined provide potential for parasitism to have a substantial impact on methane intensity (kgCH_4_/kg meat). Given that the direct effect was measured only at peak parasite infection, further research is needed to quantify the methane production of livestock throughout the infection lifecycle. The effects described here demonstrate that livestock parasitism may significantly contribute to methane emissions from livestock, suggesting that disease control could play a role in mitigating methane production from livestock.

## 4. Exploiting Sickness Behaviours for Parasite Prevention and Control

In tracing the knock-on consequences of animal behaviours in relation to environmental distributions of parasites and the behaviours associated with parasitism, we have highlighted the sensitivity of animal behaviour to parasites and parasitism. However, this sensitivity of behaviour may offer opportunities to identify infection and thus directly contribute to disease control.

Sickness behaviours are a collective suite of behavioural changes and can include decreases in overall activity, food and water intake and grooming behaviours. They are exhibited by infected individuals across a wide range of taxa in response to parasite infection [37,58,59,60,61,62] and are thought to be associated with energy conservation by the host to mount an effective immune response [34,60,63], or to promote host tolerance to infection [60,64,65,66].

Early detection of behaviour change that can be associated with GI parasitism has the potential to be used as a non-invasive tool to identify and treat only infected individuals, reducing anthelmintic use in domestic livestock systems [67]. Targeted control strategies have been proven to reduce the impact of parasitism and slow down the rate of development of resistance in livestock [68,69]. These methods aim to treat only individuals within a group based on a biological indicator of infection [67,70]. A number of indicators have been used to identify infected animals, including faecal egg counts, anaemia, dag scores, body condition and reduced weight gains [67,71]. Although it is possible to identify infection using these methods, the time taken between identifying the infected sheep and retrieving and testing the sample means there has already been a loss in production and a reduction in the welfare of the animals [72]. Thus, there is an interest within the agricultural industry in using behaviour change as a non-invasive early indicator of infection that could be used to identify and only treat animals exhibiting behavioural signals of parasitism. Furthermore, as most animals may not show clinical signs of disease during the early stages of GI nematode infection [73], there is potential for behavioural indicators to identify infected animals before the animal progresses into a clinical stage of infection, thus improving the animal welfare and the success of parasite management programs. However, the exploitation of sickness behaviours as an early indicator of parasitism depends on an ability to reliably detect a parasite-induced change in behaviour. Although sickness behaviours could enhance the chance of recovery, animals often vary their expression of sickness behaviour in order to depict other behaviours that confer a greater fitness benefit, such as increased reproductive effort [74] and increased parental effort [75]. Furthermore, for gregarious species, a reduction in sickness behaviours has been reported in favour of social behaviours, which may facilitate social position, mating opportunities and associated fitness benefits of group living [76]. Many of our grazing livestock species are social species, and the expression of sickness behaviour such as reduced activity could reduce the rate of social interactions in these herd animals, and thus any associated benefits of living in a group. Furthermore, groups are made up of individual members whose behaviour can influence the dynamics and behaviour of the whole group. Parasitism is also often overdispersed within groups, meaning not all individuals will be of the same infection status within a socially interacting group [77]. Thus, while there is evidence that parasitism can affect activity [14,21], the group dynamics and the parasitic status of individuals within that group could affect the host’s behavioural response to parasitism. These effects of parasitism have the potential to impact both parasitised and non-parasitised members in positive and negative ways [78]. This in turn may affect the ability to use the expression of sickness behaviour as an early identification of parasitised grazing livestock.

**Figure 3:**
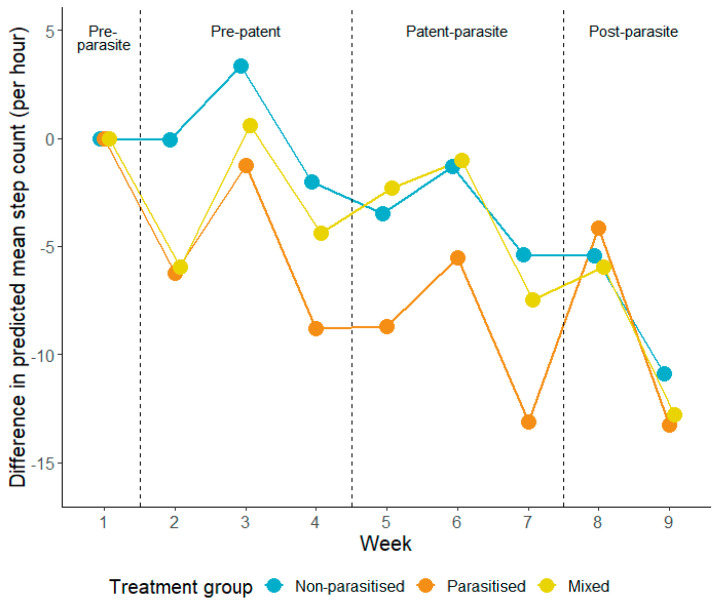
Difference in predicted mean step count per hour for non-parasitised (blue; *n* = 4), parasitised (orange; *n* = 4) and mixed (yellow; *n* = 4)) during each week of the study compared to the pre-parasite phase (week 1). The dashed lines separate the experiment into the four phases (pre-parasite, pre-patent, patent-parasite and post-parasite) (graph created by Alex M. M. Morris, from [79]).

The sensitivity of detecting sickness behaviour in relation to group dynamics of sheep was studied by [79], who investigated how the infection status of a social group impacted the activity of lambs in response to infection. This study used single parasitic-state groups of lambs (parasitised and non-parasitised) and mixed parasitic-state groups (some individuals within the group were parasitised) of lambs to investigate the expression of sickness behaviour in relation to the social context of the group. It was demonstrated that parasite-induced behavioural change was modulated by social context, with infected lambs in the mixed parasitic-state groups exhibiting reduced activity in response to the infection, to a lesser degree relative to infected lambs in the fully parasitised groups (Figure 3). However, despite this variation in the expression of sickness behaviour, this study also showed that the activity behaviour was sensitive to infection across all groups with infected lambs showing reduced activity levels immediately after parasite exposure. These behaviour changes occurred earlier than any other biological indicator of parasitism, such as faecal egg counts or changes in liveweight, which were apparent 4 weeks later. Furthermore, sickness behaviour such as reduced activity can be identified using remote monitoring systems such as accelerometers. These findings show that there is a behaviour change that can be associated with gastrointestinal parasitism in lambs that could be exploited for use in a commercial setting. Monitoring of activity behaviour could provide detailed information about specific animals within a group and could also be used to target infected individuals based on a reduction in activity levels. Thus, the results of this study demonstrate that activity-related sickness behaviours offer a promising flag for early infection in grazing livestock helping to identify and treat animals before infection intensifies, reducing welfare implications and clearing infection before production losses are realised and before animals start shedding eggs limiting contamination on pasture [79]. However, to effectively utilise parasite-induced sickness behaviour as an early indicator of disease in livestock environments, further research is required. This research should aim to understand the variability in expression of sickness behaviour across different livestock scenarios, and to validate the use of such behaviour in targeted selective treatment approaches for parasite management.

## 5. Conclusions

Animal behaviour is a key driver of parasite transmission and the resulting patterns of parasitism reduce livestock production efficiency and increase GHG pollution. However, livestock behaviour is sensitive to parasitism and has the potential to be used to identify infected animals for treatment before significant production and pollution costs are realised. By tracing the knock-on consequences of animal behaviour within a disease system, we highlight the role and importance of parasite control in livestock agriculture. While food security relies on ruminant livestock in many parts of the world, their contribution to climate change cannot be ignored. Therefore, our understanding of host–parasite interactions becomes instrumental in mitigating the consequences of these challenges. Effectively managing parasites not only enhances livestock production but also aids in methane mitigation, thereby addressing both food security concerns and climate change challenges simultaneously.

## Data Availability

Data sharing is not applicable as no new data were created or analysed in this review.
